# Perspectives From Care Seekers and Non-Care Seekers on Challenges and Recommendations to Improve Dermatologic Care in Puerto Rico

**DOI:** 10.7759/cureus.105656

**Published:** 2026-03-22

**Authors:** Joan M Neptune Rosa, Rychel Torres Rodríguez, Valeria Torres García, Camila Fontané Hoyos

**Affiliations:** 1 Department of Medicine, San Juan VA Caribbean Healthcare System, San Juan, PRI; 2 School of Medicine, Universidad Central del Caribe, Bayamón, PRI; 3 School of Medicine, Ponce Health Sciences University, Ponce, PRI; 4 Deparment of Dermatology, University of Puerto Rico, San Juan, PRI

**Keywords:** dermatologic care access, dermatology workforce, healthcare disparities, health literacy gaps, health-seeking behavior, informational barriers, patient perspective, specialist availability, structural barriers, underserved populations

## Abstract

Introduction

Barriers to dermatologic care remain a persistent challenge in Puerto Rico, where specialist shortages, long wait times, and high poverty rates limit access. While these obstacles have been described among patients who seek care, the perspectives of individuals who do not attempt to seek dermatologic evaluation remain underexplored.

Methods

We conducted a cross-sectional survey of adults in Puerto Rico recruited through community events and social media. The 18-item questionnaire assessed insurance type, care-seeking behavior within the past five years, perceived barriers to care, and recommendations for improving access.

Results

Among 456 respondents, 329 (72.1%) sought dermatologic care, while 127 (27.9%) did not. Non-care seeking was most common among uninsured respondents, with eight of 11 (72.7%) indicating that they had not sought dermatologic care, compared with 26 of 78 (33.3%) publicly insured participants and 93 of 367 (25.3%) privately insured participants (χ² = 13.34; p = 0.0013). Among non-care seekers, lack of perceived need was the most frequently reported reason for not pursuing dermatologic care (87/127; 68.5%), although many respondents also selected additional barriers. Beyond perceived need, non-care seekers and care seekers converged in identifying dermatologist scarcity and prolonged wait times as the most prominent obstacles. Subsequent barriers differed, with care seekers more commonly reporting difficulty contacting office staff to schedule appointments (165/329; 50%) and non-care seekers more often citing limited knowledge of where to seek care (19/127; 15.0%). In recommending solutions, both groups prioritized increasing the number of specialists, reducing wait time for appointments, and improving insurance coverage. Non-care seekers additionally emphasized raising awareness about skin health, while care seekers stressed improvements in scheduling processes.

Conclusion

These findings illustrate how structural shortages intersect with informational gaps, particularly among uninsured populations. Addressing these challenges through workforce expansion, streamlined scheduling systems, broader insurance participation, and culturally adapted education will be essential for achieving equitable dermatologic care across Puerto Rico.

## Introduction

Skin conditions ranging from chronic inflammatory disorders to malignancies require timely diagnosis and specialist care to achieve optimal outcomes, particularly considering that cutaneous findings may serve as early indicators of underlying systemic diseases. Access to dermatologic services, however, remains constrained by provider shortages, geographic maldistribution, prolonged wait times, and financial barriers [1-2]. These challenges disproportionately affect racial and ethnic minorities, low-income populations, and residents of underserved regions, reflecting both structural barriers, such as insurance restrictions and workforce scarcity, and informational barriers, including low-risk perception and misconceptions about skin health [3-6]. Distinguishing between these domains is essential, as they may differentially influence care-seeking behavior and require tailored intervention strategies.

Local evidence suggests that similar care limitations exist in Puerto Rico. Prior work has documented prolonged wait times for dermatology appointments and systemic obstacles to specialty care, with implications for disease progression and patient well-being [7]. These determinants are compounded by socioeconomic constraints, as a substantial proportion of the island’s population lives below the federal poverty level and relies on public insurance for health coverage [8].

Broader health system pressures likely intensify these barriers. Between 2010 and 2020, Puerto Rico experienced a marked decline in its physician workforce alongside rapid population aging, factors known to increase demand for specialty care while constraining service capacity. Reduced federal Medicaid financing relative to the United States and ongoing physician migration to the mainland further restrict access to medical services, contributing to delays in specialty care [9].

Despite these limitations, most existing research in Puerto Rico has focused on individuals already engaged with the healthcare system, leaving the experiences of those who do not seek dermatologic care largely unexplored. Accordingly, this study is designed as a descriptive assessment of perceived barriers to dermatologic care, rather than an analysis of causal relationships. By examining care challenges reported by both care seekers and individuals who refrain from pursuing dermatologic evaluation, this study aims to provide a more comprehensive understanding of structural and informational or perceptual access limitations in Puerto Rico and to inform the development of targeted, multilevel interventions to improve timely and equitable dermatologic care.

## Materials and methods

Study design and population

This cross-sectional study was conducted between March and May 2025 and included adults residing in Puerto Rico. Eligible participants were individuals aged 21 years or older who reported either having sought dermatologic care within the past five years or having not sought dermatologic care during that time, independent of perceived need or access barriers.

Participants were excluded if they were younger than 21 years of age, did not reside in Puerto Rico permanently (including temporary visitors, seasonal residents, or individuals on short-term stays), or were unable to complete the survey due to language barriers or cognitive limitations, even with assistance. Surveys with more than 90% missing responses were excluded from analysis.

The study was approved by the Institutional Review Board of Universidad Central del Caribe (approval no. 2025-21), which granted a waiver of full informed consent. Participants were provided with an electronic information sheet describing the study purpose and procedures and were required to indicate agreement prior to beginning the questionnaire. Participation was voluntary, and all responses were collected in a de-identified manner. The survey was available in both Spanish and English to accommodate participants’ language preferences.

Recruitment and data collection

Participants were recruited using a convenience-based sampling approach through community health events, local organizations, and social media advertisements, including Facebook and Instagram. Recruitment materials directed interested individuals to a secure online survey hosted on REDCap, where eligibility screening and study information were provided prior to participation.

Survey instrument and data management

A structured 18-item questionnaire was created specifically for this study to assess dermatologic care-seeking behaviors, perceived barriers to care, and recommendations to improve access. The instrument was not adapted from any previously published or copyrighted surveys; therefore, no permissions were required. The questionnaire was developed by the research team in collaboration with a board-certified dermatologist to ensure clinical relevance and subsequently reviewed by an additional board-certified dermatologist and a PhD-trained researcher with expertise in survey methodology to enhance content validity and clarity. The instrument was pilot tested among a small group of participants (n = 8) to evaluate comprehension and flow, resulting in minor refinements prior to distribution. The full survey instrument is provided in the Appendix.

The questionnaire was organized into domains addressing participant demographics, prior dermatologic care utilization, experiences accessing care, perceived barriers, and suggested system-level recommendations for improvements. Most items were multiple-choice, with several allowing respondents to select more than one option. Select questions included open-ended response options (“Other, please specify”) to capture perspectives not represented among predefined choices. Survey items were presented in a fixed order for all participants. The instrument took approximately 10 minutes to complete.

Study data were collected and managed using REDCap electronic data capture tools hosted at Universidad Central del Caribe. REDCap is a secure, web-based platform that supports research data collection through validated entry workflows, audit trails, and automated export to statistical software for analysis [10-11]. Responses were collected anonymously, and all data were de-identified prior to analysis.

Statistical analysis

Descriptive statistics were used to summarize demographic characteristics, frequencies of reported barriers, and recommended improvements. Categorical variables were reported as counts and percentages. Open-ended responses were reviewed and categorized thematically when applicable; responses that did not fit predefined categories were retained as free-text descriptors and reported descriptively. A chi-square test of independence was performed to examine the association between insurance status (private, public, uninsured) and dermatologic care-seeking behavior, with statistical significance defined as p < 0.05. All analyses were conducted using standard statistical software.

## Results

**Figure 1:**
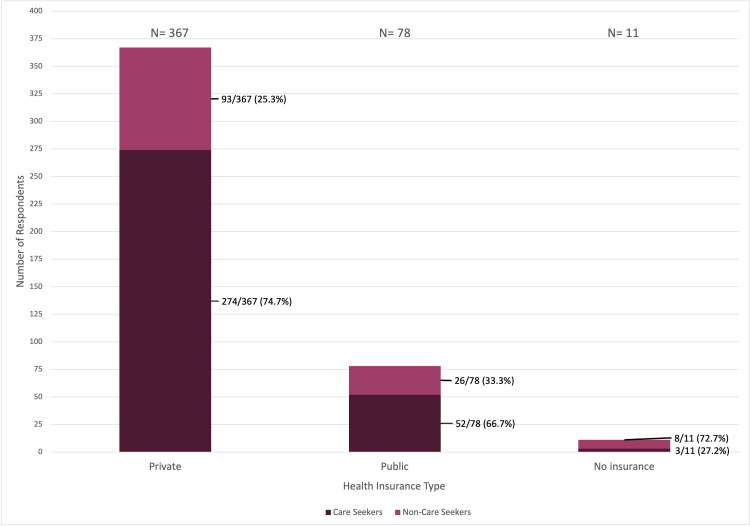
Distribution of care-seeking status by health insurance type Stacked bar chart showing the number and proportion of respondents who reported seeking versus not seeking dermatologic care within the past five years, stratified by insurance type (private, public, or uninsured). Percentages displayed within each bar represent the proportion of respondents within each insurance category, and total respondents per category are shown above each bar. Care-seeking behavior differed significantly by insurance status (χ² = 13.34, p = 0.0013).

A total of 456 individuals completed the survey; 329 (72.1%) reported seeking dermatologic care within the past five years, whereas 127 (27.9%) did not. Care-seeking behavior differed significantly by insurance status (χ² = 13.34, p = 0.0013) (Figure 1).

Among respondents who reported being uninsured (n = 11), 8/11 (72.7%) indicated no dermatologic care seeking. In comparison, lack of care seeking was reported by 26/78 (33.3%) of those with public insurance and 93/367 (25.3%) of those with private insurance.

Across respondents who did not seek dermatologic care (N = 127), the most frequently selected reason was lack of perceived need for evaluation, reported by 87/127 (68.5%). For clarity of visual presentation, responses indicating no perceived need were not displayed in the comparative barriers figure, as these respondents also selected additional barriers; these data were retained in the overall analysis and are reported in the text.

Excluding lack of perceived need, the most frequently reported barriers in the non-care-seeking group were limited dermatologist availability (21/127; 16.5%) and long wait times for appointments (21/127; 16.5%). Other commonly cited obstacles included lack of knowledge about where to seek care (19/127; 15.0%) and the perception that the condition was not severe enough to warrant evaluation (14/127; 11.0%).

**Figure 2:**
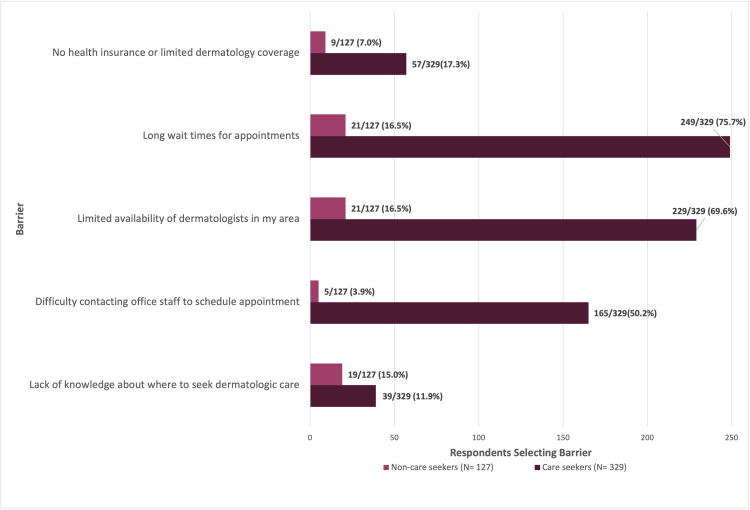
Reported barriers to accessing dermatologic care among care seekers and non-care seekers. Horizontal bar chart showing barriers to accessing dermatologic care, stratified by care-seeking status (non–care seekers, N = 127; care seekers, N = 329). Values are displayed as n/N (%), with percentages representing the proportion of respondents within each care-seeking subgroup who selected a given barrier. Participants were permitted to select multiple responses; therefore, percentages do not sum to 100%. For visual clarity, only the most frequently reported barriers are shown. “Lack of perceived need” (reported by 87/127 (68.5%) of non-care seekers) is not displayed to facilitate comparison across the remaining barriers. Complete results for all response options are provided in Appendix 2.

In care seekers (N = 329), the most frequently reported barriers were long wait times for appointments (249/329; 75.7%) and limited availability of dermatologists (229/329; 69.6%). Additional reported obstacles included difficulty contacting office staff to schedule appointments (165/329; 50.2%) and lack of health insurance or limited dermatology service coverage (57/329; 17.3%) (Figure 2).

**Figure 3:**
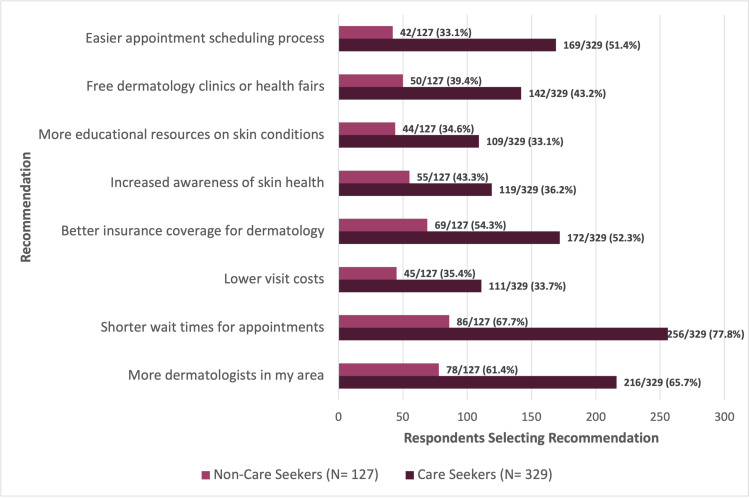
Respondent-reported recommendations to improve access to dermatologic care among care seekers and non-care seekers. Horizontal bar chart showing recommendations to improve access to dermatologic care, stratified by care-seeking status (non–care seekers, N = 127; care seekers, N = 329). Values are displayed as n/N (%), with percentages representing the proportion of respondents within each care-seeking subgroup who selected each recommendation. Participants were permitted to select multiple responses; therefore, percentages do not sum to 100%. For visual clarity, only the most frequently selected recommendations are shown; all response options and corresponding results are presented in Appendix 2.

Recommendations to improve access were closely aligned across groups. Increasing dermatologist availability was prioritized by 216 of 329 care seekers (65.7%) and 78 of 127 non-care seekers (61.4%), while reducing wait times was endorsed by 256 of 329 care seekers (77.8%) and 86 of 127 non-care seekers (67.7%). Expanding insurance coverage was similarly selected by 172 of 329 care seekers (52.3%) and 69 of 127 non-care seekers (54.3%). Divergence emerged at the next priority: easier appointment scheduling was favored by 169 of 329 care seekers (51.4%), whereas greater awareness of skin health was emphasized by 55 of 127 non-care seekers (43.3%) (Figure 3). 

Percentages for subgroup analyses were calculated using the number of respondents within each care-seeking group as the denominator. For barrier and recommendation items, participants were permitted to select multiple responses; therefore, percentages do not sum to 100%. In addition, comparisons were descriptive, and no formal statistical testing was conducted. All response options and corresponding results are presented in Appendix 2, while Figures 2-3 display only the most frequently selected items to improve visual interpretability. 

## Discussion

This study provides one of the first comparative assessments of dermatologic access barriers in Puerto Rico by capturing perspectives from individuals who sought dermatologic care and those who did not. Inclusion of non-care seekers revealed a notable insight: more than two-thirds did not perceive a need for dermatologic care, indicating that barriers to access extend beyond system capacity and include factors influencing recognition of the need for specialty evaluation. 

Comparable patterns have been reported in mainland United States populations, particularly among underserved and minority groups, where limited dermatologic knowledge has been associated with reduced engagement in skin health [12]. Prior survey-based work involving predominantly Hispanic and immigrant adults identified low awareness of skin cancer, including melanoma, along with misconceptions regarding risk among individuals with darker skin tones. Preventive practices were similarly limited, with many participants reporting infrequent skin self-examinations and low rates of prior dermatology consultation, despite expressing interest in learning more about skin cancer prevention [12].

Evidence from qualitative research offers additional context for these findings. Focus group analyses on Hispanic populations have shown that low prioritization of skin health, competing socioeconomic demands, financial constraints, and cultural norms contribute to delayed or absent preventive care [6]. For example, participants frequently reported that skin cancer is not perceived as an immediate concern compared to day-to-day responsibilities such as financial stability, leading to preventive behaviors being deprioritized. Misperceptions, such as the belief that individuals with darker skin are at low or no risk for skin cancer, further reduce perceived susceptibility and urgency for evaluation. In addition, reliance on home or “natural” remedies (e.g., aloe vera or lemon-based treatments) and concerns about sunscreen safety or cost may reinforce the perception that formal dermatologic care is unnecessary. Cultural norms related to skin tone and appearance, including preferences for tanning or beliefs about attractiveness, may also normalize sun exposure and reduce engagement in preventive practices [6].

These observations align with our finding that lack of perceived need is a dominant barrier among non-care seekers in Puerto Rico, indicating that culturally and socially mediated beliefs about risk, priority, and appropriate care may shape care-seeking behavior alongside structural constraints. Because these influences were not fully captured by the survey instrument, this represents an important limitation and supports the need for future qualitative and mixed-methods research.

Among respondents who did not seek dermatologic care, reported barriers showed substantial overlap with those described by care seekers. Regardless of perceived need, long wait times for appointments and limited availability of dermatologists were the most frequently cited obstacles, indicating a shared recognition of system-level constraints once access was considered. Differences emerged in subsequent barriers: care seekers more often emphasized operational challenges, particularly difficulty contacting office staff, whereas non-care seekers more frequently reported uncertainty about where to seek dermatologic services (Figure 2). This divergence highlights how structural and knowledge-related barriers intersect differently depending on an individual’s engagement with the healthcare system.

Our findings are consistent with prior work by Rivera-Rivera et al., which identified prolonged wait times, limited appointment availability, and difficulty contacting dermatology clinics as major barriers to care in Puerto Rico [7]. In this study, one quarter of participants reported contacting multiple clinics before securing an appointment, with delays frequently exceeding six months. Such delays were associated with worsening skin conditions, increased self-medication, and negative effects on quality of life and emotional well-being [7], underscoring the persistent and clinically consequential nature of access limitations on the island.

Insurance stratification further highlights inequities in access. Uninsured individuals were disproportionately represented among non-care seekers, while private insurance was associated with higher rates of dermatologic care seeking (Figure 1). This pattern mirrors national evidence showing that uninsured and publicly insured patients experience longer delays and reduced appointment availability [13-14]. In Puerto Rico, where poverty rates are high and public insurance predominates, these structural constraints likely compound existing disparities in both access and outcomes.

When asked to identify potential solutions, both groups converged on the same top three priorities: increasing dermatologist availability, reducing wait times for appointments, and improving insurance coverage. A contrast was observed at the fourth-ranked recommendation. Individuals already navigating the healthcare system emphasized the need for more efficient appointment scheduling, whereas non-care seekers more frequently prioritized efforts to increase skin health awareness (Figure 3). This pattern emphasizes the need for a dual approach that combines system-level expansion and operational improvements for those engaged in care with community-based education and navigation strategies for individuals not yet seeking services.

Expanding the dermatology workforce and incentivizing participation in public insurance programs are important steps, but these efforts should be complemented by collaboration with primary care clinicians, nurses, and community health workers to support prevention, triage, and early recognition of skin disease. Prior work has demonstrated that nurse-led dermatologic care can improve access, reduce disease severity, and enhance patient understanding of skin conditions, particularly in underserved settings [15]. Teledermatology may further help mitigate long wait times and geographic barriers by expanding access to specialty care, especially among publicly insured, rural, and resource-limited populations [16]. However, this modality should complement rather than replace in-person evaluation, particularly for conditions requiring procedural assessment or longitudinal follow-up. Finally, culturally tailored educational initiatives remain critical, as prior studies have shown high interest in skin health education among underserved populations when information is delivered through accessible formats such as video and text-based outreach [9].

Workforce expansion, although essential, is likely limited in the short term by structural constraints in Puerto Rico, including funding limitations and a restricted training pipeline, with a relatively small number of dermatologists serving a large population and only two residency programs producing approximately two to three specialists each year.

Teledermatology may offer a more immediately feasible and scalable approach, particularly for high-volume, low-complexity conditions, by facilitating remote triage and improving system efficiency while maintaining comparable clinical outcomes. However, its implementation depends on infrastructure readiness, including broadband access, digital literacy, and integration into existing care systems, which may pose challenges in rural and underserved areas. Similarly, nurse-led care models may help extend dermatologic capacity, though their success relies on appropriate training, regulatory support, and coordination within existing healthcare structures.

Together, these findings support a conceptual framework in which structural capacity, patient-level factors, and care navigation jointly shape dermatologic care-seeking, highlighting the need for coordinated interventions to improve equitable access in Puerto Rico.

Limitations

Limitations of this study include its cross-sectional design, which limits assessment of temporal relationships or causal inferences between perceived barriers and care-seeking behavior. Data was self-reported and therefore subject to recall bias and misclassification. Although the survey was distributed across all health districts in Puerto Rico, recruitment relied in part on community events and social media platforms, and no targeted oversampling of difficult-to-reach populations was performed. As an online-only survey, participation may have been limited among uninsured and underserved groups due to barriers such as reduced access to digital devices, internet connectivity, and lower levels of engagement with online platforms. This may have resulted in the underrepresentation of populations most affected by access barriers.

In addition, the number of uninsured respondents was relatively small, which may limit the precision of subgroup comparisons by insurance status. Analyses were not adjusted for potential confounders, including age, education, and geographic residence, and should therefore be interpreted as unadjusted. Despite these limitations, the study provides meaningful insight into both structural and perceptual barriers to dermatologic care in Puerto Rico and identifies key areas for targeted, multilevel interventions.

Recommendations

Future implementation-focused and longitudinal studies should evaluate the impact of targeted interventions using key measurable outcomes, including reductions in appointment wait times and time from symptom onset to dermatologic evaluation, earlier stage at diagnosis of dermatologic conditions (such as melanoma Breslow depth or disease severity indices), improvements in patient-reported outcomes including quality of life and satisfaction, and increased rates of timely care utilization and treatment adherence. Such an approach is essential to promote earlier engagement, reduce delays in diagnosis, and improve dermatologic outcomes across diverse populations on the island.

## Conclusions

This study demonstrates that barriers to dermatologic services in Puerto Rico are multifaceted and affect both individuals who seek care and those who do not. Across groups, limited availability of dermatologists, prolonged wait times for appointments, and insurance-related challenges consistently emerged as the most prominent obstacles, underscoring persistent system-level constraints. Notably, a substantial proportion of non-care seekers did not perceive a need for dermatologic evaluation, indicating that access limitations are shaped not only by structural factors but also by gaps in awareness and perceived relevance of skin health.

The convergence of priorities around workforce availability and appointment timeliness highlights clear targets for system-level interventions, while differences in secondary priorities, operational efficiency among care seekers, and education among non-care seekers suggest that strategies should be tailored to patients’ level of engagement with the healthcare system. Addressing dermatologic access in Puerto Rico will require coordinated efforts that pair capacity expansion and operational reforms with community-based education and navigation initiatives. Such an approach is essential to promote earlier engagement, reduce delays in diagnosis, and improve dermatologic outcomes across diverse populations on the island.

## Appendices

Appendix 1 

**Table 1:** Survey instrument assessing dermatologic care-seeking behaviors, perceived barriers, and recommendations to improve access in Puerto Rico This table presents the 18-item questionnaire used in the study, which collected information on participant demographics, dermatologic care–seeking behaviors, perceived barriers to accessing dermatologic services, and recommended strategies to improve access in Puerto Rico. Most items were multiple-choice, with several allowing multiple selections. Select questions included open-ended response options (“Other, please specify”) to capture additional perspectives not listed among predefined responses.

Section	Question no.	Question	Response options
Section 1. Demographics	1	How old are you?	21–24 years
			25–34 years
			35–44 years
			45–54 years
			55–64 years
			65–74 years
			75–80 years
			80 years or older
	2	What is your biological sex?	Female
			Male
			Intersex
			Prefer not to disclose
	3	In which municipality do you live?	All 78 municipalities of Puerto Rico including island municipalities of Vieques and Culebra
	4	What is your income level?	Less than $10,000 per year
			$10,000–$24,999 per year
			$25,000–$49,999 per year
			$50,000–$74,999 per year
			More than $75,000 per year
	5	What type of health insurance do you have?	Public (e.g., Medicaid, Medicare)
			Private
			No insurance
Section 2. Dermatologic Care History	6	Have you tried to access dermatologic care within the past five years?	Yes
			No
Section 3. Experiences Accessing Dermatologic Care (for respondents answering “Yes” to Q6)	7	What barriers did you encounter when seeking dermatologic care? (Select all that apply)	Lack of knowledge on where to seek dermatological care
			Difficulty contacting office staff to schedule an appointment
			Limited availability of dermatologists in my area
			Long wait times for appointment
			Distance to the nearest dermatologist was too far
			Financial limitations (I could not afford the visit)
			Lack of health insurance or limited dermatology coverage
			Concerns about COVID-19 or other health risks
			Difficulty finding a dermatologist who speaks my preferred language
			I prefer to manage my skin issues independently (e.g., with over-the-counter products)
			Social stigma or embarrassment related to the condition
			Other (please specify):
			I did not encounter any barriers
	8	If a dermatologist were available in your municipality, did you visit them?	Yes
			No
			Not applicable
	9	If you did not visit the dermatologist in your municipality, why? (Select all that apply)	Long wait times for appointment
			Financial constraints (could not afford the visit)
			Lack of health insurance coverage
			Did not think the condition was serious enough
			Prefer to visit a dermatologist in another city
			Not accepting patients
			Not accepting my health insurance
			Other (please specify): ___
			Does not apply (No dermatologist in my town)
			I visited the dermatologist in my town
	10	If you traveled to another city, how long was your travel time?	Less than 30 minutes
			30 minutes–1 hour
			More than 1 hour–2 hours
			More than 2 hours
			I did not travel to another city
	11	What was the waiting time for your last dermatology appointment?	Less than one week
			One to two weeks
			Three to four weeks
			Between one month to two months
			Between three months to six months
			More than six months
			Did not obtain appointment
	12	If you waited longer than one month, did you seek another healthcare provider?	Primary care physician office
			Emergency clinic
			Urgent care
			Consult at a hospital
			Telemedicine
			Other (please specify):
			Did not seek another healthcare professional
Section 4. Reasons for Not Seeking Care (for respondents answering “No” to Q6)	13	Why did you not seek dermatologic care? (Select all that apply)	I did not perceive a need
			Lack of knowledge on where to seek dermatological care
			Difficulty contacting office staff to schedule an appointment
			Limited availability of dermatologists in my area
			Long wait times for appointments
			The distance to the nearest dermatologist was too far
			Financial limitations (I could not afford the visit)
			Lack of health insurance or limited dermatology coverage
			Concerns about COVID-19 or other health risks
			Difficulty finding a dermatologist who speaks my preferred language
			I prefer to manage my skin issues independently (e.g., with over-the-counter products)
			The condition did not seem serious enough to seek specialist care
			Social stigma or embarrassment related to the condition
			Other (please specify):
			I did not encounter any barriers
	14	If dermatologic care became more accessible, would you seek care?	Yes
			No
			Maybe
Section 5. Condition-Specific Information	15	What skin conditions have you experienced in the past five years? (Select all that apply)	Acne
			Psoriasis
			Skin Cancer
			Rosacea
			Atopic dermatitis
			Contact dermatitis
			Fungal infections
			Hyperpigmentation
			Vitiligo
			Other (please specify):
			I don’t have a diagnosis
			Prefer not to disclose
			None
	16	How would you describe the severity of your skin condition?	Asymptomatic
			Mild
			Moderate
			Severe
			Does not apply
Section 6. Overall Experience and Recommendations with Dermatologic care in Puerto Rico	17	How would you rate your overall experience accessing dermatologic care in Puerto Rico?	Excellent
			Good
			Fair
			Poor
	18	What would improve access to dermatologic care in Puerto Rico? (Select all that apply)	More dermatologists in my area
			Shorter waiting times for appointments
			Lower costs of visit
			Better health insurance coverage for dermatology
			Increased awareness of skin health
			More educational resources about skin conditions
			Free dermatology clinics or health fairs
			Easier process for scheduling appointments
			Telehealth options to reduce travel
			Dermatologists who speak my preferred language
			Increased COVID-19 safety measures
			Other (please specify):

Appendix 2 

**Table 2:** Reported barriers to accessing dermatologic care among non-care seekers and care seekers Barriers to accessing dermatologic care reported by survey respondents, stratified by care-seeking status (non–care seekers, N = 127; care seekers, N = 329). Values are presented as n (%), where percentages represent the proportion of respondents within each care-seeking group who selected a given barrier. Participants were permitted to select multiple responses; therefore, percentages do not sum to 100%. “Not applicable” indicates barriers that were not assessed for care seekers.

Barrier	Care seeking status	
	Non-care seekers (N = 127)	Care seekers (N = 329)
	n (%)	n (%)
I did not perceive a need for care.	87 (68.5)	Not applicable
Lack of knowledge about where to seek dermatological care.	19 (15.0)	39 (11.9)
Difficulty contacting office staff to schedule an appointment.	5 (3.9)	165 (50.2)
Limited availability of dermatologists in my area.	21 (16.5)	229 (69.6)
Long wait times for appointments.	21 (16.5)	249 (75.7)
The distance to the nearest dermatologist was too far.	4 (3.1)	42 (12.8)
Financial limitations (I could not afford the visit).	7 (5.5)	40 (12.2)
Lack of health insurance or limited dermatology coverage.	9 (7.1)	57 (17.3)
Concerns about COVID-19 or other health risks.	1 (0.8)	4 (1.2)
Difficulty finding a dermatologist who speaks my preferred language.	0 (0.0)	0 (0.0)
I prefer to manage my skin issues independently (e.g., with over-the-counter products).	5 (3.9)	2 (0.6)
Social stigma or embarrassment related to the condition.	0 (0.0)	8 (2.4)
The condition did not seem serious enough to seek specialist care.	14 (11.0)	Not applicable
Other (please specify)	4 (3.1)	14 (4.3)
Did not encounter any barrier.	0 (0.0)	13 (4.0)

**Table 3:** Reported recommendations to improve access to dermatologic care among non-care seekers and care seekers Recommendations to improve access to dermatologic care reported by survey respondents, stratified by care-seeking status (non–care seekers, N = 127; care seekers, N = 329). Values are presented as n (%), where percentages represent the proportion of respondents within each care-seeking group who selected a given recommendation. Participants were permitted to select multiple responses; therefore, percentages do not sum to 100%.

Recommendation	Care seeking status	
	Non-care seekers (N= 127)	Care seekers (N= 329)
	n (%)	n (%)
More dermatologists in my area	78 (61.4)	216 (65.7)
Shorter wait times for appointments	86 (67.7)	256 (77.8)
Lower visit costs	45 (35.4)	111 (33.7)
Better insurance coverage for dermatology	69 (54.3)	172 (52.3)
Increased awareness of skin health	55 (43.3)	119 (36.2)
More educational resources on skin conditions	44 (34.6)	109 (33.1)
Free dermatology clinics or health fairs	50 (39.4)	142 (43.2)
Easier appointment scheduling process	42 (33.1)	169 (51.4)
Other (please specify)	1 (0.8)	12 (3.6)
